# Silver Sneakers in Central PA: Assessment of a Community based Exercise Program in a Mixed Rural/Urban Catchment Area

**DOI:** 10.1093/geroni/igab046.2890

**Published:** 2021-12-17

**Authors:** Eileen Flores, Sage Nakagawa, Robinn Moyer, Shirley Bluethmann

**Affiliations:** 1 Penn State College of Medicine, Hershey, Pennsylvania, United States; 2 Queen's University, Ontario, Ontario, Canada

## Abstract

Older cancer survivors present with unique challenges that may impact quality of life and increase physical dysfunction if not properly managed. Regular physical activity (PA) can help mitigate these effects. Silver Sneakers (SS), a free exercise program available to Medicare beneficiaries, has more than 16,000 US locations. To understand capacity of SS to serve older adults in our mixed rural/urban catchment area of Central Pennsylvania, we 1) identified all registered SS program locations in our 28-county catchment area and; 2) conducted phone questionnaires with SS program staff. Approximately 18 gyms closed during the pandemic, leaving a sample of 121 participating gyms. We talked to 80 gyms (66% response rate) to understand member and programming characteristics, training of staff and program marketing. Geographic locations of SS were mixed – 39% in rural and 61% in urban counties; the majority (43%) were located in private gyms or YMCAs. The majority of gyms reported membership was equally mixed by gender and described ages of members as 65-80 years (94%). Program staff said that many members exercised several times per week with friends/family. Program staff also reported that social opportunities (35%) were a primary reason participants remained active in SS. Most (89%) of the facilities were still able to offer SS during the pandemic, with the majority (60%) adapting format to Zoom and other video platforms to conduct classes. Overall, SS programs offer a sustainable option to facilitate access to exercise programs and reduce barriers to PA among older adults in our catchment area.

